# Characterization of the complete chloroplast genome sequence of *Tinospora sagittata* and its phylogenetic implications

**DOI:** 10.1080/23802359.2019.1698374

**Published:** 2019-12-13

**Authors:** Cuiying Peng, Junsheng Liang, Qiaoying Xie, Xujun Wang, Wei Guo

**Affiliations:** a Hunan Academy of Forestry, Changsha, Hunan, P. R. China;; b College of Forestry, Central South University of Forestry and Technology, Changsha, Hunan, P. R. China;; c Taishan Academy of Forestry Sciences, Taian, Shandong, P. R. China

**Keywords:** *Tinospora sagittata*, chloroplast genome, Illumina sequencing

## Abstract

*Tinospora sagittata* is a perennial vine of the family Menispermaceae and distributed in Hunan, Hubei, Guangxi, and Sichuan province of P. R. China. It has been used in Chinese traditional medicine for centuries. The chloroplast (cp) genome of *T. sagittata*, characterized using Illumina technology, is 163,662 bp in size. There are a total of 130 genes, coding for 85 proteins, 37 tRNAs, and 8 rRNAs. Phylogenetic relationship analysis based on 16 complete cp genome sequences exhibited that *T. sagittata* was phylogenetically closer to *Menispermum dauricum* and *Stephania japonica*.

*Tinospora sagittata* is a perennial vine of the family Menispermaceae and distributed in Hunan, Hubei, Guangxi, and Sichuan province of P. R. China. It grows in thin forests and crevices beside streams. In Chinese traditional medicine, its yellow tuberous roots, also known as ‘Jinguolan’ were widely used to treat carbuncle, furuncle, and sore throat (Institute of Botany, Chinese Academy of Sciences 1994). Primarily, most research on *T. sagittata* has revolved around phytochemical constituents (Shi et al. 2008; Li et al. 2012, 2017). It was reported that a clerodane furanoditerpenoid isolated from *T. sagittata* had cytotoxic activity against human MCF-7 and HL-60 cell lines (Qin et al. 2015). Accordingly, expanding the genomic information resources is required to adopt a creative strategy to take the best advantage of *T. sagittata*. We report here its first cp genome sequences characterized using Illumina technology.

Fully expanded leaves of *T. sagittata* were harvested from a single tree at Yuhua, Changsha, Hunan province, P. R. China (28°06′N, 113°01′E). After being rinsed using sterile water, the voucher specimen (accession no. FJG_2016_QND_WqlHu) was quickly frozen by liquid nitrogen and deposited at Hunan Academy of Forestry. About 2 μg purified genomic DNA extracted using CTAB method was sheared by sonication to produce 350 bp fragments to construct a library. Raw data were generated with the Illumina Hiseq system. After cleaning and trimming, 21,989,236 of 150 bp paired-end (PE) reads were obtained and mapped to the reference database created from all plants cp genomes downloaded from GenBank database using bowtie2 (Langmead and Salzberg 2012) with default parameters. The mapped reads were selected for cp genome assembly. Genome annotation of *T. sagittata* was completed using BLAST searches, then submitted to GenBank (MN646885) after adjusting manually.

The size of the *T. sagittata* cp genome is 163,662 bp. It contains one small single-copy (SSC, 20,193 bp) region, one pair of inverted repeat (IR, 25,366 bp each) regions, and one large single-copy (LSC, 92,737 bp) region. The DNA A + T contents of the SSC, LSC, IR regions and the whole genome are 67.52, 64.40, 56.56, and 62.35%, respectively. It is apparent that the A + T content of the two IR regions is lower than that of others. In the cp genome, there are a total of 130 genes, coding for 85 protein, 37 tRNAs, and 8 rRNAs. There are 13 intron-bearing genes, of which two genes (*clpP* and *ycf3*) harbor two introns and others harbor one intron each.

To determine the phylogenetic position of *T. sagittata*, maximum likelihood (ML) method was utilized for phylogenomic analysis. The cp genome sequences of *T. sagittata* were aligned using MAFFT v7.2 with cp genome sequences of 15 species in the Menispermaceae, Lardizabalaceae, Eupteleaceae, Berberidaceae, Papaveroideae, and Ranunculaceae families. The ML tree presented that *T. sagittata* was phylogenetically closer to the two species in the family Menispermaceae, *Menispermum dauricum* and *Stephania japonica* (Figure 1).

## Figures and Tables

**Figure 1.**
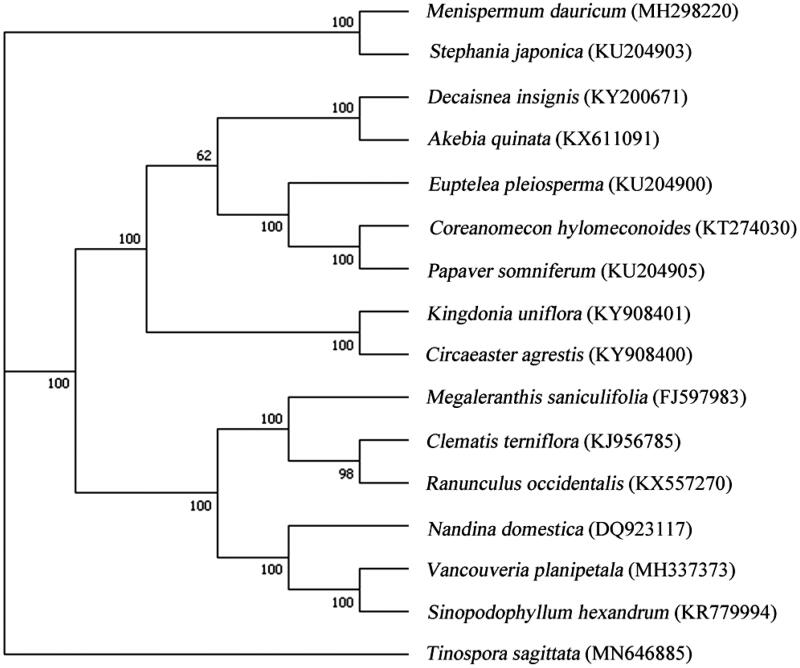
Phylogenetic tree based on 16 complete cp genome sequences. The bootstrap support values are shown next to the branches.
